# Distinct Transposable Element Transcript Patterns in Microglia Across Aging and Alzheimer's Disease

**DOI:** 10.1111/acel.70653

**Published:** 2026-08-12

**Authors:** Randy A. Grant, Rachel L. Doser, Thomas J. LaRocca

**Affiliations:** ^1^ Department of Health & Exercise Science Colorado State University Fort Collins Colorado USA; ^2^ Center for Healthy Aging Colorado State University Fort Collins Colorado USA; ^3^ Molecular, Cellular, & Integrative Neuroscience Program Colorado State University Fort Collins Colorado USA

## Abstract

Microglia, the brain's resident immune cells, are transcriptionally diverse and highly dynamic, but during aging and disease they lose their transcriptomic flexibility and adopt a chronically activated state that is associated with neuroinflammation and pathology. An emerging transcriptomic process that is also increasingly implicated in brain aging, neuroinflammation, and disease is the dysregulation of transposable elements (TEs), repetitive genomic sequences with the potential to cause cellular stress/dysfunction. However, there are limited data on microglial TE transcript patterns in these contexts. Here, we analyzed multiple RNA‐seq datasets from isolated human and mouse microglia across aging, Alzheimer's disease (AD), and AD‐associated pathology. In contrast to previous observations based on whole‐brain tissue and other brain cell types, we found that microglial TE transcript levels remained relatively consistent throughout most of the human lifespan before increasing in late life. We also found that TE transcript levels in microglia from AD patients showed minimal changes compared to age‐matched controls, and in RNA‐seq analyses of transgenic AD mouse models we observed pathology‐associated TE transcript decreases. Subsequent analyses identified inverse associations between TE transcript levels and autophagy/lysosome‐related gene expression, and in vitro studies suggested that aging‐ and AD‐relevant stimuli, as well as pharmacological autophagy inhibition, modulate TE transcript expression in cultured human microglia. Together, these data provide novel insight into TE transcript dynamics in microglia, highlighting TE transcript patterns that differ from those observed in whole‐brain samples and other cell types in aging and AD.

## Graphical abstract

Microglia‐specific transposable element patterns across human aging, Alzheimer's disease, mouse models of Alzheimer's disease pathology, and in vitro experiments.

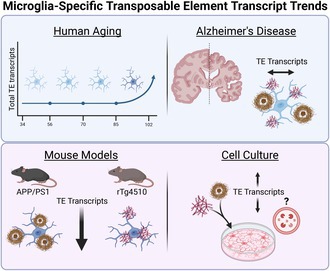

## 1 Introduction

Aging is the primary risk factor for most neurodegenerative diseases, including Alzheimer's disease (AD) (Hou et al. 2019). As such, understanding the age‐associated cellular and molecular processes that contribute to AD is an important research priority. Although the underlying mechanisms of brain aging and neurodegeneration overlap (Mattson and Arumugam 2018; Wilson et al. 2023), neurodegenerative diseases (including AD) involve cell type‐specific transcriptional changes (Lopes et al. 2022; Matusova et al. 2023; Su et al. 2022) that lead to intercellular signaling cascades that drive disease progression (Balusu et al. 2023; Ke and Gibson 2004; Srinivasan et al. 2016; De Strooper and Karran 2016). Notably, these events may be particularly relevant in microglia, the brain's primary immune cells. Microglia are particularly transcriptionally diverse, exhibiting multiple transcriptional sub‐states in brain aging and AD (Von Bernhardi et al. 2015; Olah et al. 2018, 2020; Zheng et al. 2021), and recent studies suggest that changes in microglial transcriptional plasticity may play a central role in aging/AD (Lopes et al. 2022; Prater et al. 2023; Butovsky and Weiner 2018; Wendimu and Hooks 2022; Moca et al. 2022). However, one possible consequence of microglial transcriptome changes that has not been comprehensively investigated involves aberrant expression of transposable element (TE) transcripts, which are also increasingly implicated in brain aging and AD (Copley and Shorter 2023).

TEs are repetitive genomic sequences derived from ancient mobile genetic elements that make up > 50% of the human genome (Leslie 2008). Until recently, they were often overlooked in transcriptome studies, in part due to their repetitive nature, which complicates bioinformatics analyses (Copley and Shorter 2023). However, growing evidence shows that TEs play important roles in cellular processes like gene expression, transcription, cell signaling, chromatin remodeling, and even neuroinflammation (Gianfrancesco et al. 2017; Lopez‐Pajares 2016; Nishihara 2019; Saleh et al. 2019), which is a key hallmark of brain aging and AD. Additionally, TEs are reported to be involved in multiple neurodegenerative diseases including amyotrophic lateral sclerosis (Reilly et al. 2013), Huntington's disease (Floreani et al. 2022), Parkinson's disease (Gianfrancesco et al. 2017), and AD (Evering et al. 2023; Guo et al. 2018; Ochoa et al. 2023; Scopa et al. 2023). Importantly, TEs are normally epigenetically silenced, but our lab and others have shown that aging is associated with increased TE transcript levels in human peripheral (LaRocca et al. 2020) and brain (Wahl et al. 2023) tissue, and that AD and AD‐related pathological proteins (i.e., tau) exacerbate these increases (Guo et al. 2018; Ochoa et al. 2023; Wahl et al. 2023; Cavalier et al. 2025; Ramirez et al. 2022; Sun et al. 2018). Our reports and others' also suggest that TE transcript accumulation contributes to age‐ and disease‐associated inflammation, and that pharmacologically targeting TEs may be a viable therapeutic strategy (Ravel‐Godreuil et al. 2021; Wang et al. 2025; McEntee and LaRocca 2025; Smith et al. 2024). Interestingly, these age‐ and disease‐associated TE transcript increases are thought to arise from epigenetic alterations and, perhaps, reduced transcript degradation via autophagy (McEntee and LaRocca 2025; Cheray and Joseph 2018; Choi et al. 2023; Yeh and Ikezu 2019), processes that are highly dynamic in microglia (Cheray and Joseph 2018; Choi et al. 2023; Yeh and Ikezu 2019; Wang et al. 2021). Still, despite these observations and the central role of microglia in neuroinflammation and the pathophysiology of aging/AD, microglial TE dynamics in these contexts remain largely unexplored.

To provide initial insight into TE dynamics in microglia across aging and AD in the present study, we analyzed TE transcript expression in multiple RNA‐seq datasets on isolated post‐mortem human microglia. We found unexpected changes in TE transcript expression patterns, including minimal age‐related increases until late life and only modest changes in AD (as opposed to the progressive age‐related increases and AD‐related exacerbation of TEs observed in whole‐brain samples (Copley and Shorter 2023; Wahl et al. 2023; Smith et al. 2024)). Subsequent analyses of these datasets and others on transgenic mouse models suggested these effects may be associated with AD pathology (i.e., tau and amyloid beta [Aβ]), and with differences in autophagy/RNA degradation machinery gene expression. Follow‐up, preliminary in vitro experiments also suggested that aging‐ and AD‐relevant stimuli, as well as pharmacological modulation of autophagy/lysosomal pathways, may alter TE transcript levels in human microglia. Together, our results provide new insight into TE dynamics in the most active immune cell type within the aging/AD brain, as well as a basis for future studies on cellular TE regulation in general.

## 2 Methods

### 2.1 RNA‐Seq Datasets and Availability

All RNA‐seq data used in this manuscript were generated in previously published studies and are available on the Gene Expression Omnibus (GEO). Datasets included: No Cognitive Impairment (NCI) Dataset 1: human microglia isolated from cognitively normal individuals during full body autopsy (total RNA, GSE99074 (Galatro et al. 2017)), AD Dataset 2: human microglia isolated from AD patients and cognitively normal age‐matched controls during autopsy (total RNA, GSE146639 (Alsema et al. 2020)), AD Dataset 3: human microglia isolated from frozen samples of AD patients and cognitively normal age‐matched controls (total RNA, GSE125050 (Srinivasan et al. 2020)), Mouse Dataset 1: microglia isolated from WT mice across lifespan (total RNA, GSE131869 (Gyoneva et al. 2019)), Mouse Dataset 2: microglia isolated from rTg4510 mice (total RNA, GSE123467 (Wang et al. 2018)), Mouse Dataset 3: microglia isolated from APP/PS1 mice (total RNA, GSE205569 (Yin et al. 2023)).

### 2.2 Bioinformatics Analyses

For analyses of TE transcript patterns in microglia with aging and AD pathology, human and mouse TE/transcriptome data were generated from previously published, publicly available RNA‐seq datasets of isolated microglia (described above). Secondary analyses of these datasets were performed as recently reported (LaRocca et al. 2020; Wahl et al. 2021, 2023; Smith et al. 2024). Briefly, reads were trimmed and filtered with the *fastp* program (Chen et al. 2018) then mapped to the mouse (mm10) or human (hg38) genome with the STAR aligner (Dobin et al. 2013) with settings adjusted to allow for multi‐mapping of TEs (Wahl et al. 2023). Gene and TE transcript counts were generated from the aligned bam files using TEcount in the TEtranscripts program (Jin and Hammell 2018), which counts multi‐mapping reads against a repeatmasker annotation file containing all TEs (including class 1 and 2 TEs, satellite sequences, tRNAs, etc.). Gene and TE counts were analyzed together for differential expression so that TEs could be compared to overall gene/library transcript levels in R using DESeq2 software (Love et al. 2014), with significant differences determined using the default Wald test. Normalized counts and DESeq2 outputs were used for all downstream and statistical analyses.

### 2.3 HMC3 Cell Culture

For in vitro experiments, the human microglial clone 3 (HMC3) cell line was commercially obtained from ATCC and cultured in Dulbecco's Modified Eagle's Medium (HyClone) containing 10% fetal bovine serum (Gibco) and 1% penicillin/streptomycin (HyClone) at 37°C in an atmosphere containing 5% CO_2_. For experiments, cells were treated at 100% confluency with tumor necrosis factor alpha (TNF‐⍺, 300 ng/mL, Sigma‐Aldrich), Aβ_1–42_ (5 μM, Abcam), tau‐441 preformed fibrils (50 nM, rPeptide), or Chloroquine (100 nM, Cayman Chemical) for 24 h and then flash frozen for use in downstream analyses.

### 2.4 Immunoblotting

Cell samples were thawed on ice, lysed in ice‐cold RIPA buffer containing phosphatase and protease inhibitors (ThermoFisher/Roche), and 5 μg of protein was separated by electrophoresis on 4%–12% Bis‐Tris gels (Bio‐Rad). The protein was then transferred to nitrocellulose membranes (Bio‐Rad) and blocked using TBS‐Tween containing 5% BSA for 1–2 h at room temperature. Primary antibodies (interleukin 6, IL‐6; Novus Biologicals; 1:1000 dilution) were diluted in TBS‐Tween containing 5% BSA, added to membranes, and incubated overnight at 4°C. HRP‐linked secondary antibodies (Cell Signaling, 1:5000 dilution) were then diluted in TBS‐Tween containing 5% BSA, added to membranes, and incubated for 1 h at room temperature. Proteins were subsequently detected using ECL chemiluminescent substrate (ThermoFisher) on a FluorChem E imager (ProteinSimple), and protein levels (ECL signal intensities) were measured using ImageJ2/Fiji and normalized to total protein loaded as visualized with Ponceau S staining (ThermoFisher).

### 2.5 RT‐PCR

Cells were rinsed with DPBS and gently lysed in Trizol reagent. RNA was recovered and purified using an RNA‐specific spin column kit (Direct‐Zol, Zymo Research), which included DNaseI treatment to remove genomic DNA. cDNA was generated from 1 μg RNA per condition using qScript cDNA SuperMix Synthesis Reaction (Quantabio) according to manufacturer's instructions. For each PCR reaction, 5 ng cDNA was used with PerfeCTa SYBR Green FastMix, Low ROX (Quanta Biosciences) and run on an CFX Opus 96 real‐time PCR instrument. Presented data are normalized to the B2M housekeeping gene. Primers included: B2M forward: 5′‐ATGAGTATGCCTGCCGTGTGA‐3′, reverse: 5′‐GGCATCTTTCAAACCTCCATG‐3′; L1Hs 5′ end forward: 5′‐TCACCAGCAACGGAACAAAG‐3′, reverse: 5′‐TTGAATGTCCTCCCGTAGCTC‐3′; LTR5Hs forward: 5′‐GCTCTCTGAAACATGTGCTGT‐3′, reverse: 5′‐TTTGTGTCCCTGGGTACTTGAG‐3′; GSATX forward: 5′‐ACCCAGGGTGCGTGTCTC‐3′, reverse: 5′‐CTCAACGTCCCCCTGAAAGC‐3′; AluYi6 forward: 5′‐CACGCTTGTAATCCCAGCAC‐3′, reverse: 5′‐TTAGTAGAGACGGGGTTTCACC‐3′; HERVK14C‐int forward: 5′‐ACAAATTCAGCAGGCAAGGC‐3′, reverse: 5′‐AGAGCTATGATCGACACGTCTG‐3′.

### 2.6 Statistics

Differential expression analyses were performed as described above using DESeq2 software. In downstream analyses of TE transcripts, normalized counts were compared against total transcript levels using GraphPad Prism software (unpaired *t*‐test on DESeq2 normalized counts). Correlation analyses were performed using simple linear regressions (Pearson correlation) with multiple hypothesis testing corrections (Bonferroni correction), and RT‐PCR data were analyzed with one‐way ANOVA, all using GraphPad Prism software.

## 3 Results

**FIGURE 1:**
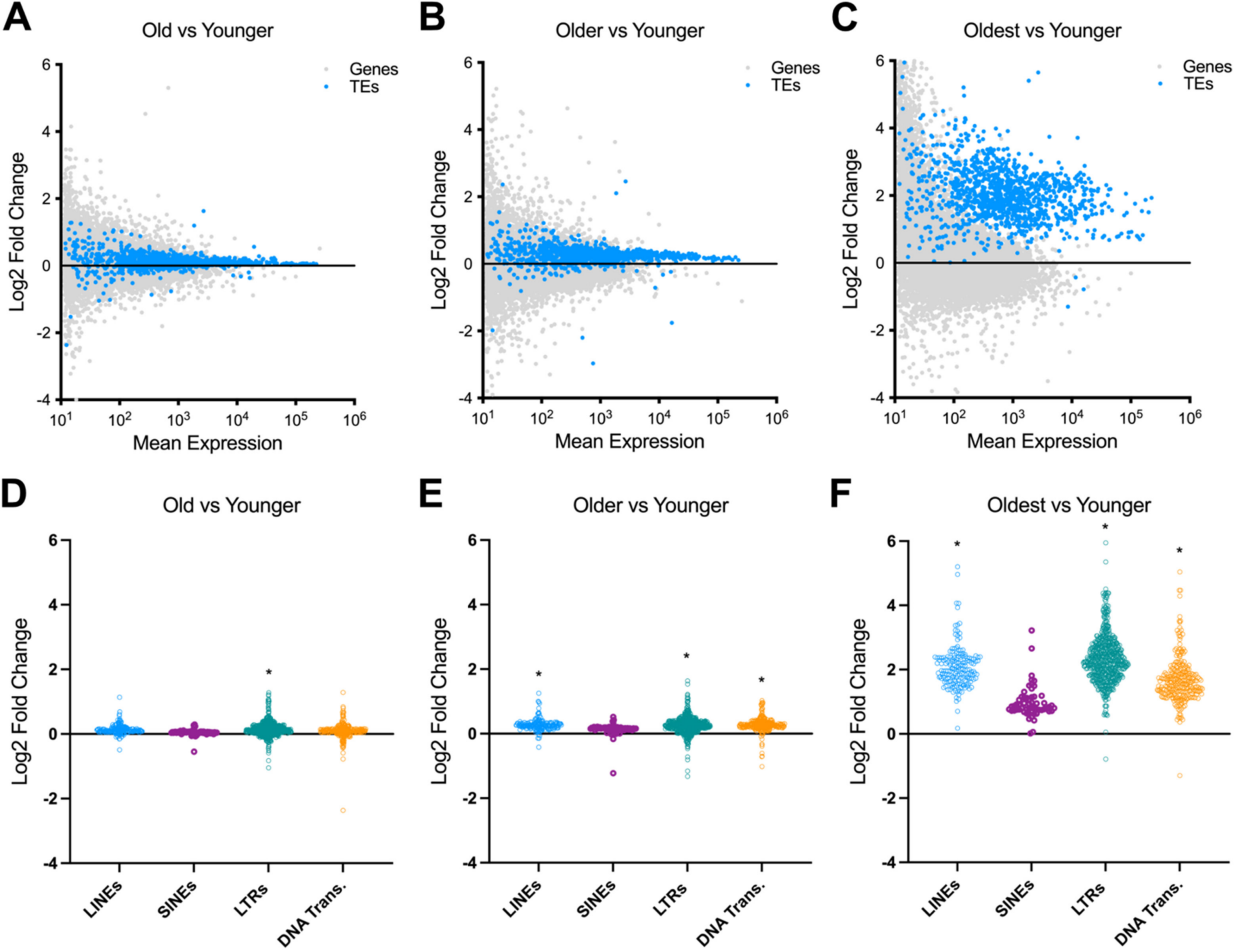
TE transcripts increase in human microglia with aging. (A–C) MA plots showing relative TE and gene transcript levels in isolated microglia from old, older, and oldest versus younger cognitively normal adults. (D–F) Relative TE transcript levels by major type in the same comparisons. Ages: Younger (34–56 years, *n* = 18), old (57–69 years, *n* = 16), older (70–84 years, *n* = 14), oldest (85–102 years, *n* = 7). *p < 0.05.

To provide initial insight into TE transcript dynamics in aging microglia, we analyzed pre‐existing publicly available RNA‐seq data on isolated microglia from cognitively normal adults, aged 34–102 years (NCI dataset 1; (Galatro et al. 2017)). Across all subjects, we observed a weak, non‐significant positive correlation between age and total normalized TE transcript levels (Figure S1A), but when we binned subjects into quartiles by age range and controlled for the effects of sex in differential expression analyses, a clearer pattern emerged. TE transcripts increased across age comparisons (Figure S1B), but compared to microglia from younger/middle‐aged subjects (aged 34–56), microglia from “old” adults (aged 57–69) showed minimal increases in TE transcript levels (0 differentially expressed TEs with an adjusted *p*‐value < 0.05), with overall trends driven primarily by LTRs (Figure 1A,D). Similarly, TE transcript levels only increased minimally (0 differentially expressed TEs with an adjusted *p*‐value < 0.05) in microglia from even “older” subjects (aged 70–84; Figure 1B,E), but in microglia from the “oldest” subjects (aged 85–102) TE transcripts greatly increased (413 differentially expressed TEs with an adjusted *p*‐value < 0.05) relative to young/middle‐aged adults (Figure 1C), despite no significant changes in total normalized gene transcript counts (Figure S1C). Interestingly, SINEs were the only major TE type that did not significantly increase in the oldest subjects compared to the younger group (Figure 1F). These findings are generally in line with data showing that TE transcript levels increase in isolated neurons (Ravel‐Godreuil et al. 2021), astrocytes (McEntee et al. 2023), and whole brain samples (Wahl et al. 2023) with aging. However, the fact that microglial TE transcript levels remained relatively stable until very old age was somewhat surprising, potentially suggesting unique features of microglia that may suppress TE transcript accumulation across the lifespan. Consistent with this idea, in similar analyses of a dataset based on isolated mouse microglia, TE transcript levels were largely unchanged in both middle‐aged and older animals (Figure S2), despite reports showing age‐related increases in whole mouse brains (Ramirez et al. 2022).

**FIGURE 2:**
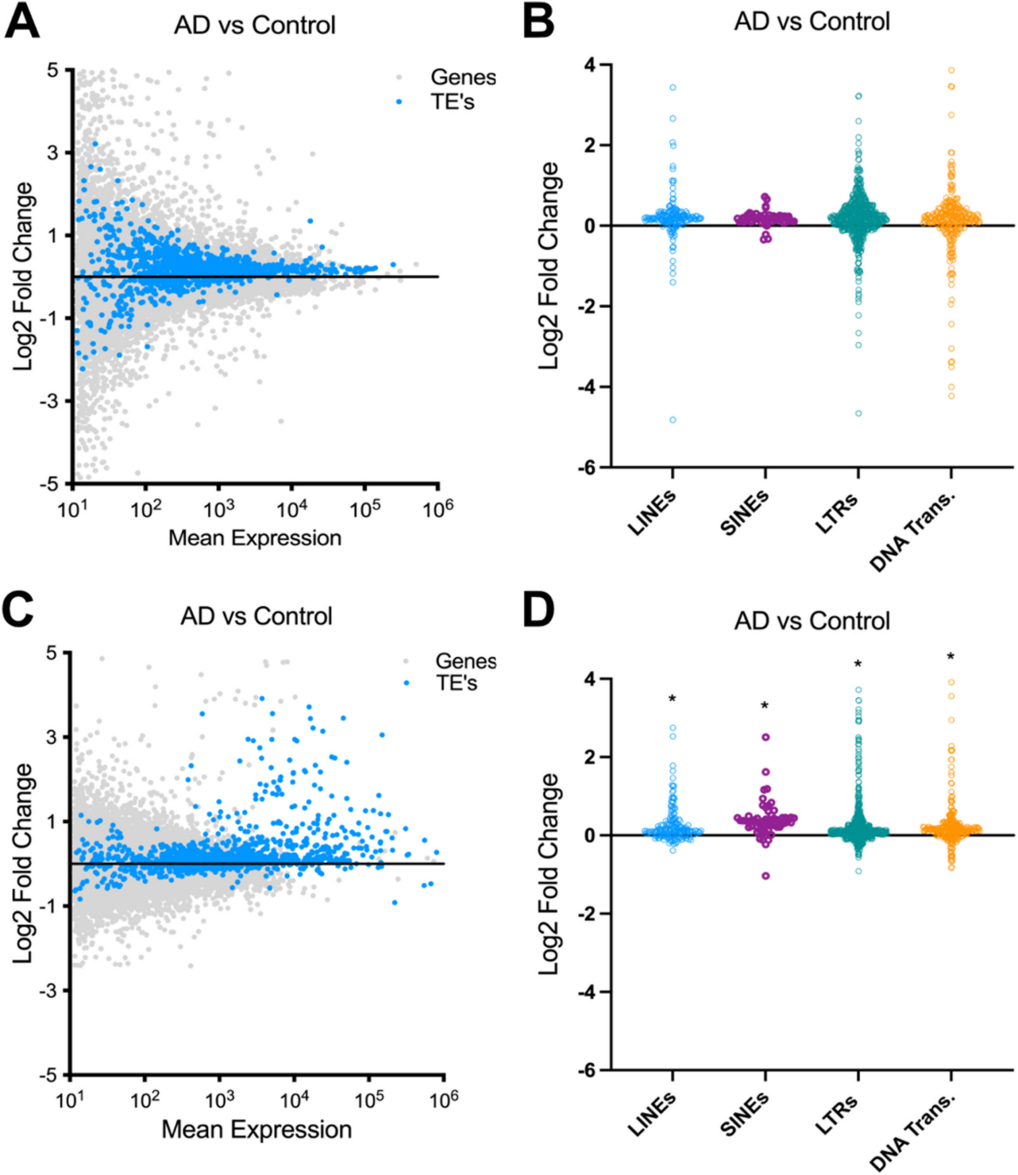
TE transcripts increase minimally in microglia from Alzheimer's disease patients. (A, C) MA plots showing relative TE and gene transcript levels in isolated microglia from Alzheimer's disease patients versus cognitively normal adults in two different datasets. (B, D) TE transcript levels by major type in the same comparisons. Ages; AD Dataset 2 (A, B): Control (55–103 years, *n* = 15), AD (59–98 years, *n* = 10). AD Dataset 3 (C, D): Control (38–90+ years, *n* = 15), AD (59–84 years, *n* = 10). *p < 0.05.

AD is reported to exacerbate age‐related TE transcript accumulation in neurons (Feng et al. 2024), astrocytes (Ochoa et al. 2023), and whole brain samples (Guo et al. 2018; Wahl et al. 2023; Sun et al. 2018). To determine how AD might impact TE expression in aged microglia, we analyzed two existing RNA‐seq datasets on microglia isolated from AD patients and cognitively normal age‐matched controls. In both datasets (AD Dataset 2 (Alsema et al. 2020) and AD Dataset 3 (Srinivasan et al. 2020)), we found that total normalized TE transcript levels showed no correlation with age (Figure S3A,D) and were largely unchanged in AD microglia compared to controls (Figure S3B,E). Similarly, in covariate‐adjusted differential expression analyses, TE transcript levels were minimally increased (0 differentially expressed TEs with an adjusted *p*‐value < 0.05 in either dataset) in AD patient microglia compared to controls (Figure 2A,C). All TE subtypes followed the same pattern as overall TE transcript levels (Figure 2B,D), with no differences in total normalized gene transcript counts (Figure S3C,F). This lack of consistent or strong TE transcript increases in isolated AD microglia contrasts with data in previous reports from other cell types, in which TE transcripts consistently and markedly increase with AD. Thus, these results further suggested that TE transcripts may be uniquely regulated in microglia compared to other brain cell types in the context of AD and neurodegeneration.

**FIGURE 3:**
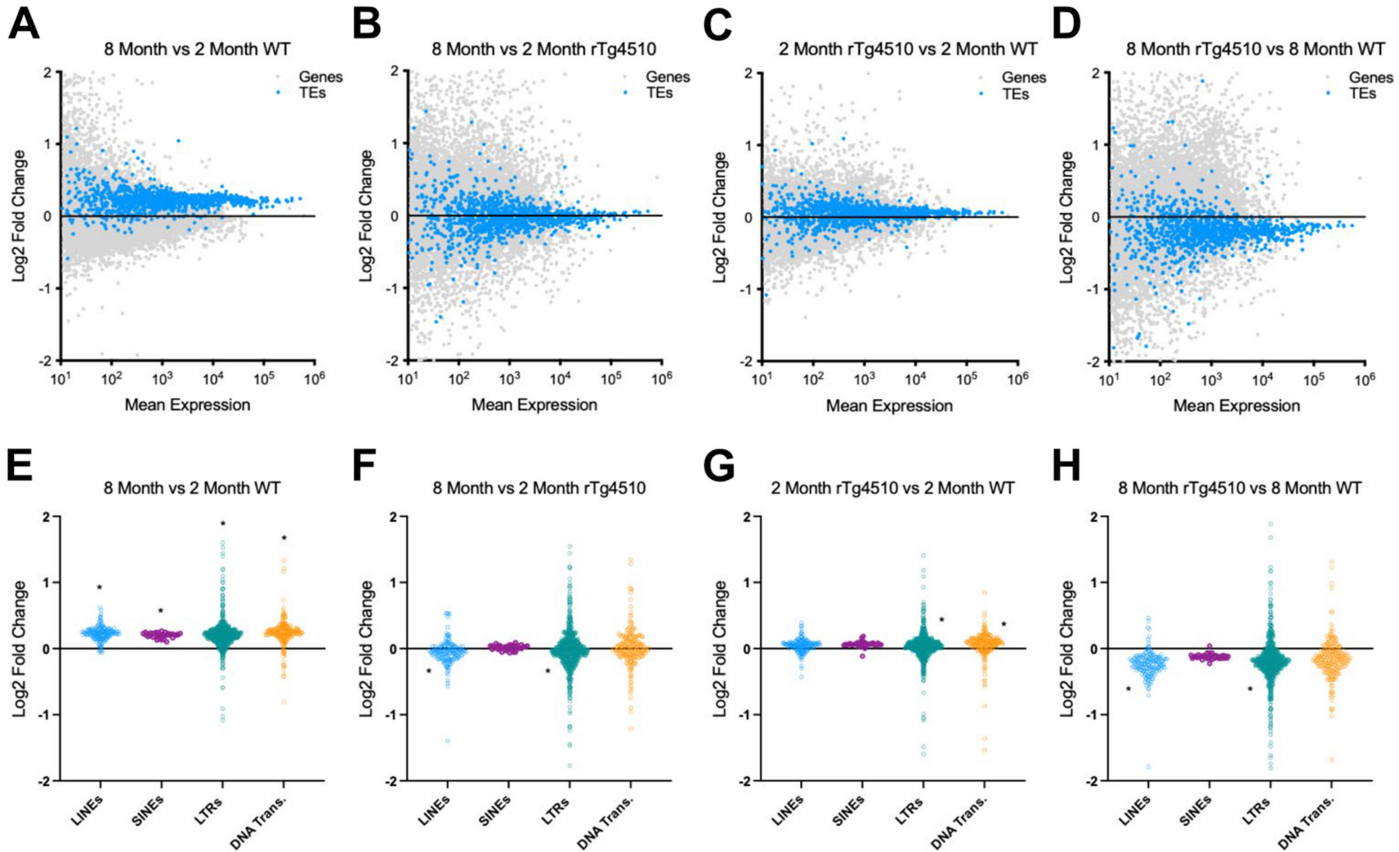
TE transcripts decrease in rTg4510 mouse microglia. (A–D) MA plots showing gene and TE transcript expression patterns in isolated microglia from rTg4510 mice and WT littermate‐controls at 2 and 8 months old. (E–H) Matched comparisons showing relative levels of TE transcripts by major type. Sample size: 2 month (WT *n* = 12, rTg4510 *n* = 12), 8 month (WT *n* = 12, rTg4510 *n* = 12). *p < 0.05.

Given our observations above and the central role of microglia in responding to AD pathology, we hypothesized that the absence of major TE transcript increases in AD microglia may be linked to pathological proteins. To address this, we analyzed existing RNA‐seq data on isolated microglia from rTg4510 tauopathy mice and APP/PS1 amyloid beta (Aβ) pathology mice. We found that normalized average TE counts of both APP/PS1 and rTg4510 mouse microglia were significantly reduced compared to wildtype littermate controls (Figure S4A,B). In differential expression analyses, TE transcript levels increased only slightly in microglia from 8‐month‐old compared to 2‐month‐old wildtype/littermate control mice (Figure 3A,E), similar to the subtle age‐related increases we observed in cognitively healthy “older” humans. However, in microglia from 8‐month‐old versus 2‐month‐old rTg4510/tau mice, TE levels were somewhat reduced compared to total transcript levels (Figure 3B), and this was driven by significant decreases in LINEs and LTRs (Figure 3F). Interestingly, in 2‐month‐old rTg4510 mice compared to 2‐month‐old wildtype/littermate control mice, TE transcript levels were moderately increased (278 differentially expressed TEs with an adjusted *p*‐value < 0.05), with overall trends driven mainly by LTRs and DNA transposons (Figure 3C,G). However, when comparing 8‐month‐old rTg4510/tau mice to 8‐month‐old wildtype mice, we observed strong reductions (580 differentially expressed TEs with an adjusted *p*‐value < 0.05) in TE expression, primarily driven by decreases in LINEs and LTRs (Figure 3D,H).

**FIGURE 4:**
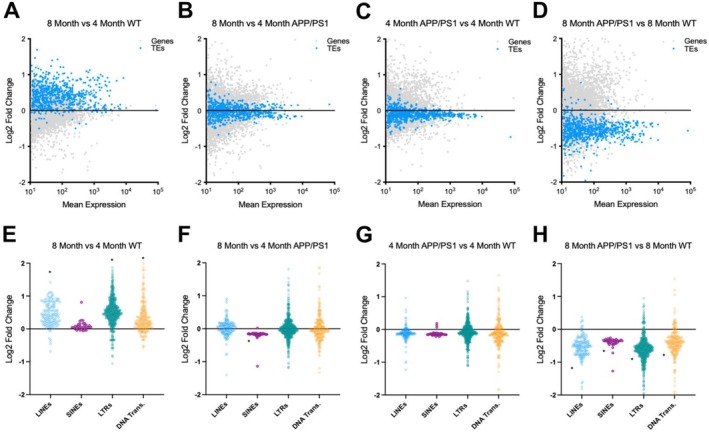
TE transcripts decrease in APP/PS1 mouse microglia. (A–D) MA plots showing gene and TE transcript expression patterns in isolated microglia from APP/PS1 mice and WT littermate‐controls at 4 and 8 months old. (E–H) Matched comparisons showing relative levels of TE transcripts separated by major type. Sample size: 4 month (WT *n* = 24, APP/PS1 *n* = 20), 8 month (WT *n* = 24, APP/PS1 *n* = 22). *p < 0.05.

Our results above were somewhat unexpected, given previous reports showing that tau causes TE transcript increases in whole brains and isolated neurons (Guo et al. 2018; Ochoa et al. 2023; Ramirez et al. 2022; Sun et al. 2018; Grundman et al. 2021). However, similar to our findings in rTg4510/tau mice, in a second dataset based on isolated microglia from APP/PS1 mice (Aβ pathology model), we again observed pathology‐associated decreases in TE transcripts. Specifically, we found an overall increase in TE transcripts in 8‐month‐old compared to 4‐month‐old wildtype/littermate control mice (Figure 4A,E), but the same aging comparison in APP/PS1 mice revealed minimal overall differences (Figure 4B), with significant reductions in SINEs (Figure 4F). Moreover, when comparing 4‐month‐old APP/PS1 mice directly to 4‐month‐old wildtype/littermate control mice, we found that TE transcript levels were moderately reduced (11 differentially expressed TEs with an adjusted *p*‐value < 0.05) (Figure 4C,G). Interestingly, in 8‐month‐old APP/PS1 mice compared to 8‐month‐old wildtype/littermate control mice, we observed marked TE transcript decreases (618 differentially expressed TEs with an adjusted *p*‐value < 0.05) both overall and by major type (Figure 4D,H). These findings potentially suggest a contribution of both tau and Aβ to the distinct TE transcript patterns observed in pathology‐affected microglia. Also, as our lab and others have reported whole‐brain TE transcript increases in aged rTg4510/tau mice (Wahl et al. 2023; Ramirez et al. 2022), these results further point towards microglia‐specific, and perhaps pathology‐related patterns, of TE transcript expression.

**FIGURE 5:**
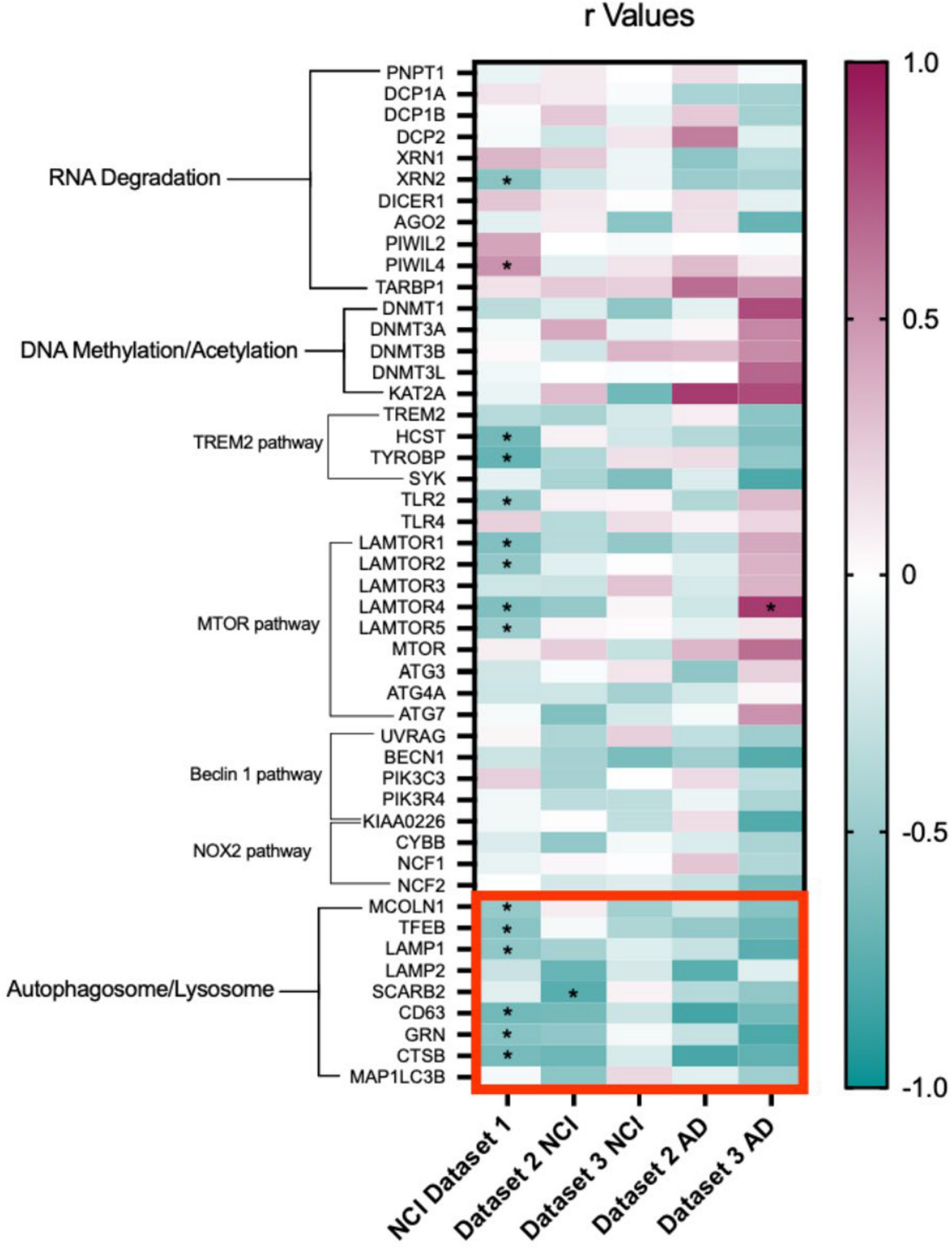
Correlations among key genes from candidate TE‐modulating processes and total normalized TE transcript levels. Heatmap showing Pearson correlations between key genes that represent candidate TE‐modulating processes and total normalized TE transcript counts in all three human datasets. *p < 0.05.

Multiple biological processes could contribute to the TE transcript decreases we observed in pathology‐associated microglia, including classical RNA degradation pathways (Bamezai et al. 2012; Lehner and Sanderson 2004; Sturm et al. 2015), epigenetic modifications (e.g., methylation and acetylation) (Rigal and Mathieu 2011; Shvedunova and Akhtar 2022), microglial metabolism/immune signaling (through multiple pathways) (Janda et al. 2018), and autophagy (which has been reported to degrade TE transcripts (Guo et al. 2014)). To provide further insight into which of these biological processes might be most involved with the TE patterns we observed, we evaluated correlations among TE transcript levels and key genes associated with each process (selected based on gene ontology membership and specificity) across all three human datasets, with NCI and AD patients analyzed separately (Figure 5). We chose this approach instead of untargeted transcriptome‐wide analyses to avoid false hits and/or the possibility of missing modest yet biologically relevant correlations with key microglial genes and processes. Interestingly, we observed several significant gene/TE correlations, but our most consistent finding was that normalized autophagy/lysosome‐related transcript levels were inversely related to total normalized TE transcript levels across datasets, suggesting this biological process may play a role in the TE transcript patterns observed in our human data analyses. Consistent with this idea, in NCI Dataset 1 we observed decreases in processes that intersect with autophagy at late age (Figure S5A–D), coinciding with the marked TE transcript increases shown in Figure 1. Correlations among total normalized TE transcripts and key autophagy/lysosome‐related genes were also present across age groups within NCI Dataset 1, the largest of all datasets analyzed (Figure S5E). Additionally, in AD Datasets 2 and 3, in which TE transcript increases were expected but not present, we observed consistent AD‐associated increases in autophagy gene expression (Figure S6). Together, these results suggest the possibility that differences in autophagy/lysosomal pathway activity could be associated with microglial TE transcript variation.

**FIGURE 6:**
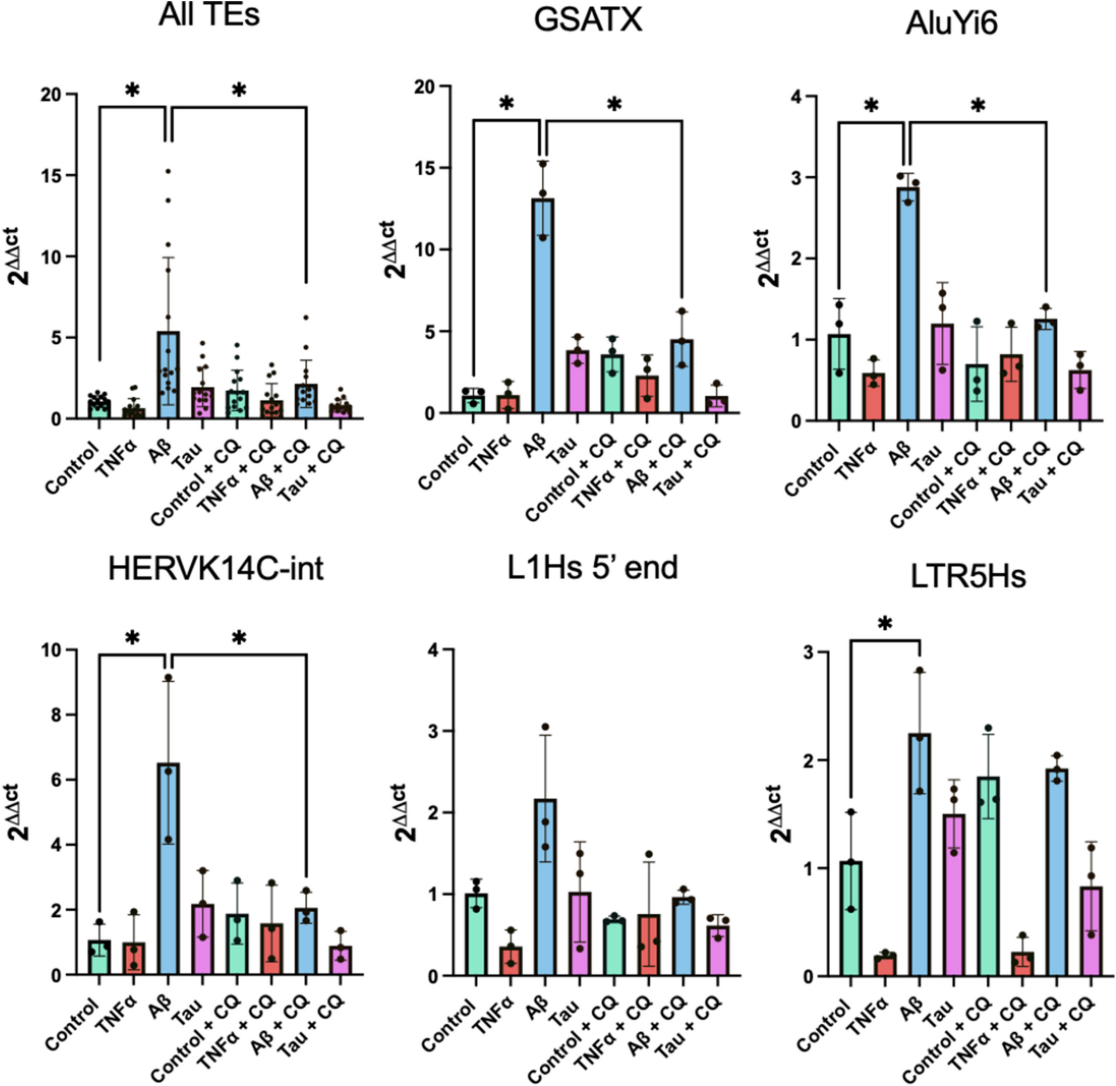
RT‐PCR analysis of key TE transcripts in HMC3 microglia exposed to aging and AD‐related pathology treatments. Cells were exposed to tumor necrosis factor (TNF‐ɑ), amyloid‐beta_1–42_ (Aβ), Tau‐441 preformed fibrils, and/or chloroquine (CQ) for 24 h prior to RNA extraction and subsequent RT‐PCR for analysis of various key TE transcript levels. *p < 0.05.

Finally, to explore the potential association between TE transcripts and autophagy in microglia with aging/AD pathology, we performed preliminary and hypothesis‐generating in vitro experiments using the well‐characterized HMC3 microglial cell line. To confirm that these cells responded as expected (i.e., like microglia), we first verified that they upregulated inflammatory cytokines in response to pro‐inflammatory stimuli (Figure S7). Then, we used RT‐PCR to measure transcript levels of five key TEs that followed the general trends in our human analyses under various aging‐ and pathology‐relevant conditions (Figure 6). Although we observed slightly different effects for each TE measured, the general pattern that emerged was as follows: First, in cells treated with the autophagy inhibitor chloroquine, we observed a non‐significant trend for TE transcript increases, consistent with previous data showing that autophagy may degrade TEs (Guo et al. 2014), and with our finding that autophagy gene expression was inversely related to TE transcript levels across the human datasets we analyzed. Second, when these cells were exposed to inflammatory cytokine treatment (TNF‐ɑ, an aging/disease‐relevant cytokine), TE transcripts did not increase and even tended to decrease in some comparisons. In contrast, when these cells were exposed to Aβ or tau, we observed increased expression of all TEs examined (although effects did not reach significance for tau), consistent with previous reports showing that tau pathology is associated with TE dysregulation. However, co‐treatment of Aβ or tau with autophagy inhibition reduced TE transcripts to levels similar to controls (and this effect was most significant in Aβ‐treated cells). This was a surprising result considering that autophagy inhibition alone tended to increase TE transcripts and our finding that autophagy gene expression was inversely related to TE levels in human AD samples. Thus, these findings do not support a simple model in which reduced autophagy increases TE transcripts in microglia per se, but they do suggest that relationships among aging/AD‐related stimuli, autophagy/lysosomal activity, and TE transcripts may be context‐dependent, which could in part explain our divergent findings in human datasets.

## 4 Discussion

Microglia are dynamic, CNS‐resident immune cells that perpetually survey the environment while adjusting their transcriptome to best support surrounding neurons and glia (Zheng et al. 2021; Young et al. 2021). They play a central role in brain aging and neurodegenerative disease (Olah et al. 2018; Gao et al. 2023; Hickman et al. 2018; Li et al. 2023; Mosher and Wyss‐Coray 2014; Song and Colonna 2018), with growing evidence suggesting that microglial transcriptional patterns reflect various stages of disease progression and pathology (Yeh and Ikezu 2019; McQuade and Blurton‐Jones 2019). Emerging data also show that TE transcripts play an important role in brain aging and neurodegenerative diseases (Copley and Shorter 2023; Saleh et al. 2019; Ravel‐Godreuil et al. 2021), including AD (Evering et al. 2023; Guo et al. 2018), but there are limited data on microglial TE expression specifically in these contexts. In the present study, we explored the relationship between brain aging, AD, and TEs in microglia by analyzing multiple pre‐existing RNA‐seq datasets on isolated human and mouse age/AD‐associated microglia. In contrast to previous reports on whole‐brain data, we showed that age‐related increases in microglial TE transcript levels are minimal across most of the human lifespan and not majorly exacerbated by AD/pathology, and that these patterns may be associated with age‐ and pathology‐related changes in RNA homeostasis and cellular quality control pathways, including autophagy/lysosomal activity. Although preliminary, these findings suggest several important points about TE homeostasis in microglia and the aging/AD brain, which we discuss in the following sections.

First, human microglia have uniquely regulated transcriptomes in aging and disease compared to other CNS cell types (Beutner et al. 2013; Gerrits et al. 2020), which may in part explain the unexpected TE transcript patterns we observed. Beyond their cell‐specific expression of the “sensome” (microglia‐specific genes that aid in sensing and reacting to their immediate microenvironment (Hickman et al. 2013)), microglia highly express many AD risk genes (Hansen et al. 2018). In fact, although microglia have been historically classified into two broad categories (homeostatic and activated), recent single‐cell RNA‐seq studies have identified as many as nine different microglia transcriptional sub‐states (Olah et al. 2020), suggesting a transcriptional continuum (Masuda et al. 2019; Yaqubi et al. 2023) that allows microglia to play a central role in various aspects of CNS homeostasis including neurogenesis, synaptic pruning, and neuroinflammation (Cheray and Joseph 2018; Paolicelli et al. 2011; Perry et al. 2010; Sato 2015; Li and Barres 2018). In aging and disease, the microenvironment surrounding microglia becomes complex and pathologically remodels the cells' chromatin landscape, leading to epigenetic and transcriptional changes that drive pathology‐associated substates (Masuda et al. 2019; Fujita and Yamashita 2021; Sankowski et al. 2019). Increased chromatin accessibility in transcriptionally active/plastic cell types allows for rapid transcriptional changes (Mansisidor and Risca 2022), and consequentially, may promote greater expression of TE transcripts as a byproduct of this heightened genomic activity. Additionally, as TEs are thought to be involved in gene regulation (Ali et al. 2021; Fueyo et al. 2022; Sundaram et al. 2014; Tossolini et al. 2025) and affected by events like read‐through transcription (Pabis et al. 2024), rapid changes in microglial transcriptional profiles could lead to greater dysregulation of TEs. These ideas would be consistent with our present findings. That is, whereas recent data suggest TE transcripts increase somewhat linearly and progressively with aging in both peripheral tissues (LaRocca et al. 2020) and whole brain samples (Wahl et al. 2023), we observed only small, non‐biologically relevant increases in TE transcripts throughout the majority of the adult lifespan, followed by marked increases in later life (85+), when aging pathology has likely accumulated in the CNS and microglia are responding accordingly (Blinkouskaya et al. 2021; Edler et al. 2021). We note that these results were all based on associations, and that cause and effect could not be determined in the retrospective analyses we performed. Additionally, RNA‐seq datasets on isolated microglia are uncommon, and there are very few single studies synthesizing data from multiple isolated microglia datasets, making the pool of data for our analyses limited. Future studies would benefit not only from additional data, but also perhaps approaches that would link microglial TE regulation with epigenetic control (e.g., chromatin or methylation sequencing approaches).

Second, the central role of microglia in sensing and modulating disease‐associated pathology may influence their TE‐related transcriptional profiles. AD, characterized by the accumulation of pathological Aβ and tau, is reported to exacerbate the TE transcript increases seen in aged whole‐brain samples (Wahl et al. 2023). However, when we analyzed two separate datasets on isolated human microglia from AD patients and cognitively normal age‐matched controls, we found that TE transcript levels in AD‐associated microglia displayed minimal changes (i.e., beyond those observed in age‐matched control subjects). One possible reason for these TE transcript patterns in AD‐associated microglia could involve pathological proteins, which accumulate in the AD brain (Alonso et al. 1996; Elobeid et al. 2016; Murphy and LeVine 2010). Indeed, pathological tau has been linked with TE transcript dysregulation in mice, drosophila, and in vitro human models (Ochoa et al. 2023; Ramirez et al. 2022; Grundman et al. 2021), but the association between Aβ and TEs is less characterized. To test this idea, we analyzed data on isolated microglia from pathological tau and Aβ mouse models. Interestingly, we found overall TE transcript decreases (that were not present at baseline in young transgenic vs. wild‐type animals) in both models of pathology. Additionally, in individual dataset comparisons, certain TE subclasses appeared to be affected more than others. However, these patterns were not consistent across datasets, and specific TEs that were most increased/decreased also varied considerably among cohorts and models. This highlights the complexity of TE regulation in aging and disease, in which microglia are the primary responders to pathology, and future studies analyzing single‐cell RNA‐seq data would be valuable for understanding how various microglia substates may drive overall TE transcript patterns in the presence of disease. Although beyond the scope of this initial study, such analyses are now possible thanks to pipelines for analyzing TEs in single‐cell data (McEntee and LaRocca 2025; Rodríguez‐Quiroz and Valdebenito‐Maturana 2022).

Third, the highly phagocytic nature of microglia, which display increases in autophagy/lysosomal activity in AD (Cho et al. 2014; D'Andrea et al. 2004; Nakanishi and Wu 2009), could provide insight into cellular processes modulating TE dynamics in general. We hypothesized that age/disease‐related changes in autophagy/lysosomal activity (which are reported to degrade TE transcripts (Ravel‐Godreuil et al. 2021)) associated with activated microglia could be one contributor to the TE transcript patterns we observed. Consistent with this possibility, we observed inverse correlations among key autophagy‐related genes and total normalized TE transcript levels across datasets, as well as trends for reduced expression of genes relevant to autophagy and other related processes with age that were somewhat reversed (increased) in AD. Although these analyses were exploratory and based on candidate genes, they suggested autophagy/lysosomal activity as a potential contributor to variations in TE transcript levels in microglia. Therefore, to further investigate this possibility, we performed preliminary hypothesis‐generating in vitro analyses in HMC3 microglia. Notably, when autophagy was inhibited in these cells, we observed a modest increase in TE transcript levels, consistent with the idea that autophagy helps to reduce TE transcript accumulation in microglia. Additionally, when we treated these cells with an aging‐relevant stimulus (TNF‐ɑ) and autophagy inhibitor, TE transcripts increased modestly (compared to TNF‐ɑ alone). This was also consistent with our human analyses showing autophagy decreases in old age while TE transcripts increase at the same timepoint. When these cells were treated with tau or Aβ, TE transcripts increased, consistent with reports of tau's effect on TEs in other cell types. To our knowledge, these are the first data to reveal similar (and perhaps even stronger) effects of TE dysregulation in microglia with Aβ exposure. The reason for these effects is unclear, although it may involve the transition of homeostatic microglia to a more activated state (which is especially robust in response to Aβ (Meda et al. 1995)), as the transcriptional plasticity required for this transition could dysregulate TE expression (Olah et al. 2020; Liang et al. 2024). Interestingly, these TE transcript increases were significantly blunted by autophagy inhibition. Again, the mechanisms underlying this observation are not clear, but microglial autophagy and Aβ clearance are tightly linked (Cho et al. 2014), so it is possible that reduced Aβ uptake due to autophagy inhibition limits Aβ‐induced effects on TEs.

Although our in vitro experiments do not establish causal mechanistic relationships, they suggest aging, AD, and autophagy‐relevant stimuli may be capable of altering TE transcript levels in human microglia. It is important to note that our experiments involved short‐term treatments (whereas AD is chronic and gradual) and blunt autophagy tools (chloroquine likely has effects beyond autophagy inhibition). Additionally, we chose to use HMC3 cells because of their tractability and because primary microglia tend to lose their phenotype in culture. However, HMC3 cells do not adopt a bona fide disease‐associated microglia (DAM) state in response to pathological insults (Rai et al. 2020; Woolf et al. 2025; Dello Russo et al. 2018). Although autophagy generally declines during brain aging (Plaza‐Zabala et al. 2017) and AD progression (Zare‐shahabadi et al. 2015), DAMs are specifically known to upregulate autophagy (Choi et al. 2023). Thus, although HMC3 cells are a well‐characterized model, the degree to which they reflect aging/AD‐associated microglia in vivo is unclear. Still, our findings support the idea that pathology and autophagy may play roles in modulating TE transcript levels in microglia (although the exact effects are likely context‐dependent), and our data may help provide insight into microglial TE dynamics and RNA homeostasis in general.

This study was intended to be an initial, hypothesis‐generating exploration of TE transcript dynamics in human microglia. Although preliminary, our collective observations from human/mouse RNA‐seq data along with our in vitro studies could suggest a model in which microglial TE transcript levels are shaped by aging, AD/pathology‐related microglial state changes, and RNA homeostasis, including autophagy/lysosomal pathways. However, our data do not support a specific causal mechanism, and additional studies are needed to determine if autophagy or other mechanisms regulate TE abundance in microglia in vivo. For example, it also is possible that TE transcripts are modified by non‐canonical RNA disposal pathways in aging/AD microglia, such as exosome release (Baixauli et al. 2014; Danzer et al. 2012; Guo et al. 2020; Pomilio et al. 2020; Cambier et al. 2021; Mohammadinasr et al. 2024), and such events could underlie the TE transcript reductions we observed in AD‐associated microglia and/or autophagy‐inhibited HMC3 microglia. As such, future studies could analyze the contents of microglia‐secreted exosomes, perhaps in the presence and absence of pathology/inhibitors to assess TE transcripts and other potential sources of endogenous inflammation, as this may provide insight into general RNA homeostasis in microglia with aging and disease. Finally, additional studies are needed to determine the extent to which the events described above may be represented in vivo, and how they might affect functional outcomes like phagocytosis. Indeed, all of our bioinformatics analyses were based on microglia that went through isolation and RNA‐sequencing preparation procedures, and our in vitro studies leveraged the HMC3 immortalized microglia line (which is well‐characterized but still different from primary cells). We also only probed for 5 key TEs in vitro, and it is possible that probing for other TEs (or conducting RNA‐seq) could lead to different insight. Uncovering such details of TE transcript dynamics in microglia, the most active immune cell type in the brain, is an important future direction, as therapeutic strategies targeting TEs will require a more complete understanding of cell type‐specific differences in their regulation.

## Author Contributions

R.A.G. designed the study, wrote the paper, generated/analyzed human and mouse TE transcript data, performed correlational analyses, and conducted all in vitro experiments (including immunoblotting and RT‐PCR). R.L.D. assisted with generating and analyzing RT‐PCR data and provided manuscript editing support. T.J.L. designed the study, wrote/edited the paper, assisted with all in vitro and transcriptome analyses, provided conceptual insight, and provided funding.

## Funding

This work was funded by the National Institutes of Health, National Institute on Aging award AG078859 (T.J.L.).

## Conflicts of Interest

The authors declare no conflicts of interest.

## Supporting information


**Figure S1:** Transposable elements with age in microglia. (A) Simple linear regression of total normalized TE transcript counts plotted against subject age. (B) Distribution of log2 fold changes for all TEs from DESeq2 differential expression analyses. (C) Normalized total transcript count for each subject grouped by age. One‐way ANOVA was used to test for significance. *p < 0.05.
**Figure S2:** TE transcripts show minimal changes in WT mice microglia with early aging. (A, B) MA plots showing relative TE and gene transcript levels at 12 and 24 month versus 2 month old WT mice. (C, D) TE transcript levels separated by major type in the same comparisons. Sample size: 2 month (*n* = 11), 12 month (*n* = 10), 24 month (*n* = 10). Unpaired *t*‐test of TE families against total transcripts was used to test for significance. *p < 0.05.
**Figure S3:** Transposable elements in microglia show minimal changes with AD. (A, D) Simple linear regressions of total normalized TE transcript counts plotted against subject age showing weak, non‐significant associations with age. (B, E) Total normalized TE transcript counts averaged across subject diagnosis. (C, F) Normalized total transcript count for each subject grouped by diagnosis. Subfigures (A–C) were generated from AD Dataset 2, while subfigures (D–F) were generated from AD Dataset 3.
**Figure S4:** Normalized average counts of all TEs in both AD mouse models. (A) Normalized average TE counts of rTg4510 mice compared against littermate controls. (B) Normalized average TE counts of APP/PS1 mice compared against littermate controls. Unpaired *t*‐test was used to test for significance. *p < 0.05.
**Figure S5:** Age‐associated changes in pathways intersecting with autophagy and their relationships with TE transcript levels. Relative expression of normalized gene counts from NCI Dataset 1 for all key genes listed in figure 5 for (A) autophagy; (B) RNA degradation; (C) mTOR; and (D) TREM2 separated by age group. Analyzed via one‐way ANOVA *p < 0.05. (E) Pearson's correlation of total normalized TE transcript levels against the gene list in Figure 5, corrected via multiple hypothesis testing.
**Figure S6:** Autophagy‐related gene expression is increased in human microglia isolated from AD patients. Normalized averaged counts for all key genes listed in Figure 5 for autophagy, RNA degradation, mTOR, and TREM2 in AD versus cognitively normal controls. (A–D) AD Dataset 2; and (E–H) AD Dataset 3. All plots were analyzed via unpaired *t*‐test. *p < 0.05.
**Figure S7:** Western blot of HMC3 cells probing for IL‐6 under various conditions. Cells were exposed to lipopolysaccharide (LPS, 10 or 100 ng/mL), interleukin‐1 beta (IL‐1β, 10 ng/mL) in conjunction with interferon gamma (IFNγ, 10 ng/mL), chloroquine (CQ, 10 μM) or a PKR inhibitor (C16, 250 nM) in conjunction with LPS, doxorubicin (Doxo, 50 nM), and tumor necrosis factor (TNF‐ɑ, 300 ng/mL) for 24 h prior to lysis and protein analysis. (A) Ponceau staining showing total protein levels added to each well. (B) Relative expression of IL‐6 in HMC3 under various conditions. (C) Image of IL‐6 western blot. One way ANOVA was used to test for relative expression significance. *p < 0.05.

## Data Availability

The data that support the findings of this study are available in Gene Expression Omnibus at https://www.ncbi.nlm.nih.gov/geo/. These data were derived from the following resources available in the public domain:—GSE99074, https://www.ncbi.nlm.nih.gov/geo/query/acc.cgi?acc=GSE99074‐GSE146639, https://www.ncbi.nlm.nih.gov/geo/query/acc.cgi?acc=GSE146639‐GSE125050, https://www.ncbi.nlm.nih.gov/geo/query/acc.cgi?acc=GSE125050‐GSE131869, https://www.ncbi.nlm.nih.gov/geo/query/acc.cgi‐GSE123467, https://www.ncbi.nlm.nih.gov/geo/query/acc.cgi?acc=GSE123467‐GSE205569, https://www.ncbi.nlm.nih.gov/geo/query/acc.cgi.
