# Supplementary material for: Distinct Transposable Element Transcript Patterns in Microglia Across Aging and Alzheimer's Disease
Source: Aging Cell. 2026 Aug 12;25(8):e70653. doi: 10.1111/acel.70653 (PMC13469183; doi:10.1111/acel.70653)
Supplement: Supplementary file 1 [file ACEL-25-e70653-s001.docx]

**SUPPLEMENTARY DATA**

**Supplementary Figure 1. Transposable elements with age in microglia. A)** Simple linear regression of total normalized TE transcript counts plotted against subject age. **B)** Distribution of log2 fold-changes for all TEs from DESeq2 differential expression analyses. **C)** Normalized total transcript count for each subject grouped by age. One-way ANOVA was used to test for significance. *P < 0.05

**Supplementary Figure 2. TE transcripts show minimal changes in WT mice microglia with early aging. A – B)** MA plots showing relative TE and gene transcript levels at 12 and 24 month vs. 2 month old WT mice. **C – D)** TE transcript levels separated by major type in the same comparisons. Sample size: 2 month (n = 11), 12 month (n = 10), 24 month (n = 10). Unpaired t-test of TE families against total transcripts was used to test for significance. *P < 0.05.

**Supplementary Figure 3. Transposable elements in microglia show minimal changes with AD. A,D)** Simple linear regressions of total normalized TE transcript counts plotted against subject age showing weak, non-significant associations with age. **B,E)** Total normalized TE transcript counts averaged across subject diagnosis. **C,F)** Normalized total transcript count for each subject grouped by diagnosis. Subfigures A – C were generated from AD Dataset 2, while subfigures D – F were generated from AD Dataset 3.

**Supplementary Figure 4. Normalized average counts of all TEs in both AD mouse models. A)** Normalized average TE counts of rTg4510 mice compared against littermate controls. **B)** Normalized average TE counts of APP/PS1 mice compared against littermate controls. Unpaired t-test was used to test for significance. *P < 0.05.

**Supplementary Figure 5. Age-associated changes in pathways intersecting with autophagy and their relationships with TE transcript levels.** Relative expression of normalized gene counts from NCI Dataset 1 for all key genes listed in figure 5 for **A)** autophagy; **B)** RNA degradation; **C)** mTOR; and **D)** TREM2 separated by age group. Analyzed via one-way ANOVA *P < 0.05. E) Pearson’s correlation of total normalized TE transcript levels against the gene list in Figure 5, corrected via multiple hypothesis testing.

**Supplementary Figure 6. Autophagy-related gene expression is increased in human microglia isolated from AD patients**. Normalized averaged counts for all key genes listed in Figure 5 for autophagy, RNA degradation, mTOR, and TREM2 in AD vs cognitively normal controls. **A-D)** AD Dataset 2; and **E-H)** AD Dataset 3. All plots were analyzed via unpaired t-test. *P < 0.05.

**Supplementary Figure 7. Western blot of HMC3 cells probing for IL-6 under various conditions.** Cells were exposed to lipopolysaccharide (LPS, 10 or 100 ng/mL), interleukin-1 beta (IL-1β, 10 ng/mL) in conjunction with interferon gamma (IFNγ, 10 ng/mL), chloroquine (CQ, 10 μM) or a PKR inhibitor (C16, 250nM) in conjunction with LPS, doxorubicin (Doxo, 50nM), and tumor necrosis factor (TNF-ɑ, 300 ng/mL) for 24 h prior to lysis and protein analysis. **A)** Ponceau staining showing total protein levels added to each well. **B)** Relative expression of IL-6 in HMC3 under various conditions. **C)** Image of IL-6 western blot. One way ANOVA was used to test for relative expression significance. *P < 0.05.
